# Verses

**Published:** 1907-07

**Authors:** 


### VERSES.

### ELBERTA GIRL AND A PEACH.

This is my taste,
Bucolic and chaste.
While the sky is bright above me,
An Elberta peach
Forever in reach
And an Elberta girl to love me.

—Newspaper.

### HIT OR MISS.

We may not have a cherry tree
On which we can make good,
Like Georgie with his hatchet,
But we can all saw wood.

—Chicago News.

### A WIFE.

We ought to be happy, egad;
In favor man's basking.
The best thing in life's to be had
For the mere asking.

—Washington Herald.

### RHYMING ENIGMA.

My first is in bag but not in string;
My second's in chain, but not in ring;
My third is in sell, but not in pay;
My fourth is in week, but not in day;
My fifth is in boy, but not in girl;
My sixth is in clean, but not in soil;
My seventh is in like, but not in hate;
My eighth is in Mabel, but not in Kate;
My whole is a favorite outdoor game,
Which often makes you boys quite lame.

—Newspaper.

### PLOWING CORN.

Gee dar Polly,
Keep een de row,
Haw now er little,
Den strate we kin go.

Bustin out middles,
Killin ob de grass,
Des wurkin de crap,
An got ter do hit fas.

Pull an swet Polly,
Hits wid you an me,
Ef de lay by time,
Bring sweet cawn ter me.

So git up Polly,
Swish yer tail an go,
An keep on er movin,
Ter de end ob de row.

—B. H. Teague.

### COURAGE.

If fate should steal your happiness
And take it far away.
And then return expectantly
To watch you weep and pray;
Just hold your head well up, dear,
And face the threatening years;
Drink the bitter cup she gives,
And smile through all your tears.

Fate knows no law or justice,
Nor cares what heart she breaks;
To him who hath enough she gives,
From him who hath nought, takes.
So run your race with lifted head,
And take things like a man;
Don't grieve if Fortune fails you,
You've done the best you can.

—Irene L. Dearing.

